# Pattern and Outcome of Thoracic Injuries in a Busy Tertiary Care Unit

**DOI:** 10.7759/cureus.11181

**Published:** 2020-10-26

**Authors:** Misauq Mazcuri, Tanveer Ahmad, Ambreen Abid, Pratikshya Thapaliya, Mansab Ali, Nadir Ali

**Affiliations:** 1 Thoracic Surgery, Jinnah Postgraduate Medical Centre, Karachi, PAK; 2 General Surgery, Jinnah Postgraduate Medical Centre, Karachi, PAK

**Keywords:** thoracic trauma, blunt chest injuries, penetrating chest injuries, hemothorax, tube thoracostomies, mortality

## Abstract

Introduction: Thoracic traumas are one of the most commonly encountered injuries in the emergency room. They range from blunt chest injuries due to road traffic accidents to penetrating chest injuries. Immediate medical and surgical interventions are essential to improve the outcome. This study was conducted to assess the pattern of thoracic trauma presenting to the emergency room, their outcome and factors contributing to it.

Methods: This prospective, observational, cross-sectional study was conducted in the Department of Thoracic Surgery, Jinnah Post Graduate Medical Center, Karachi from January 1 until July 31, 2020, with institutional ethical approval. Patients age ≥12 years presenting with traumatic thoracic injury with or without associated injuries were included. Characteristics of their injuries and management outcome were studied. All data was processed through Statistical Package for Social Sciences (SPSS) Statistics version 22 (IBM Corp., Armonk, NY, USA).

Results: A total of 199 patients were included; 154 (77.4%) patients were male and 45 (22.6%) patients were female. The most common age group presenting with trauma was the middle age (30-60 years), which included 101 (50.8%) patients. Out of the total, 126 (63.3%) had blunt chest injuries and 73 (36.6%) had penetrating chest injuries. Road traffic accidents were the most common cause of blunt chest injuries seen in 83 (65.8%) patients, whereas gunshot was the most common mode of penetrating chest injuries encountered in 41 (56.2%) cases. Tube thoracostomies were performed in 166 (83.4%) patients and thoracotomies in seven (3.51%) patients. Out of the total, 57 (28.6%) patients required mechanical ventilation and it was associated with blunt trauma, hemothorax, rib fracture, abdominal and head injuries (p ≤0.05). Mortality was seen in 22 (11.1%), which was associated with hemothorax, head injuries, mechanical ventilation and severe blood loss (p ≤0.05).

Conclusion: Traumatic thoracic injuries are a preventable cause of mortality. Blunt chest injuries are more common than penetrating chest injuries. Proper implementation of public safety measures ensures less frequent and severe outcomes. Emergency department team and specialized thoracic surgeons must come together to manage these critical patients with utmost care.

## Introduction

Trauma is among the most common reasons for hospitalization across the world. As many as two-thirds of patients of trauma have chest trauma [1]. Thoracic trauma may range from a rib fracture to life-threatening injury involving the heart or great vessels. After head injury, thoracic trauma is the second leading cause of death [1]. In the United States, 35% of all trauma-related mortalities are as a result of thoracic trauma alone or in association with other non-thoracic injuries [2].

Blunt chest injuries (BCI) are more common than penetrating and account for 20-25% of all thoracic trauma-related mortalities [2]. One of the most important causes of blunt chest injuries is road traffic accidents (RTAs), especially in developing countries [3]. RTAs constitute almost 80% of all cases of BCI. Other causes include a fall from height, assault, and occupational injuries [2]. BCI is critical and can lead to acute respiratory failure and death due to airway obstruction and lung injury [4]. Penetrating chest injuries (PCIs), although less common, can be acutely fatal. Immediate intervention is critical in PCI in order to prevent mortality. PCIs are mostly a result of assault including stab wounds, gunshot, and occupational mishaps [5].

In the Pakistani population, traumatic thoracic injuries are more common among the younger age group (21-50 years). The commonest thoracic injuries include rib fractures, with head injuries contributing the common associated non-thoracic injuries [6]. Poor outcome in patients with thoracic trauma is predicated by advanced age [2], hemothorax [7], need for mechanical ventilation (MV) [7], and associated non-thoracic injuries [8,9].

The study is conceived to quantify the burden of thoracic injuries in a large tertiary care hospital and evaluate the factors responsible for morbidity and mortality in thoracic injuries.

## Materials and methods

A prospective, observational, cross-sectional study was conducted in the Department of Thoracic Surgery, Jinnah Post Graduate Medical Center, Karachi. The duration of this study was from January 1 until July 31, 2020. The study was conducted after ethical approval from the Institutional Review Board. Informed consent was obtained from all study participants and their guardians.

Patients age 12 years or above, presenting with traumatic injury to the thorax with or without other associated injuries, were included in the study. All patients were assessed and managed according to the Advanced Trauma Life Support (ATLS) protocol. It was followed by radiological evaluation such as X-ray, Focused Assessment with Sonography in Trauma (FAST), or computed tomography (CT) scan of the brain as indicated. In the case of associated non-thoracic injuries, a multidisciplinary approach was followed. Patients were stabilized within the emergency room. Tube thoracostomies and thoracotomies were performed as indicated. Mechanical ventilation was started if required.

Patient data were collected on a questionnaire comprised of demographic information of the patient, characteristics of injury, primary diagnosis, associated thoracic and non-thoracic injuries, medical and surgical interventions, and outcome of the injury. All data was processed through Statistical Package for Social Sciences (SPSS) Statistics version 22 (IBM Corp., Armonk, NY, USA). For categorical data, frequencies and percentages were calculated. Chi-square was applied to determine statistical correlation. For continuous data, mean and standard deviation (SD) was calculated. Independent T-test was applied to determine statistical correlation. P-value ≤0.05 was taken as significant.

## Results

A total of 199 patients with thoracic trauma were included in this study, 154 (77.4%) were males and 45 (22.6%) were females. The mean age was 39.45 ± 16.46 years (range: 12-90 years). There were 62 (31.1%) young participants (age less than 30 years), 101 (50.8%) middle aged participants (age 30-60 years), and 36 (18.1%) elderly (age more than 60 years).

**Figure 1:**
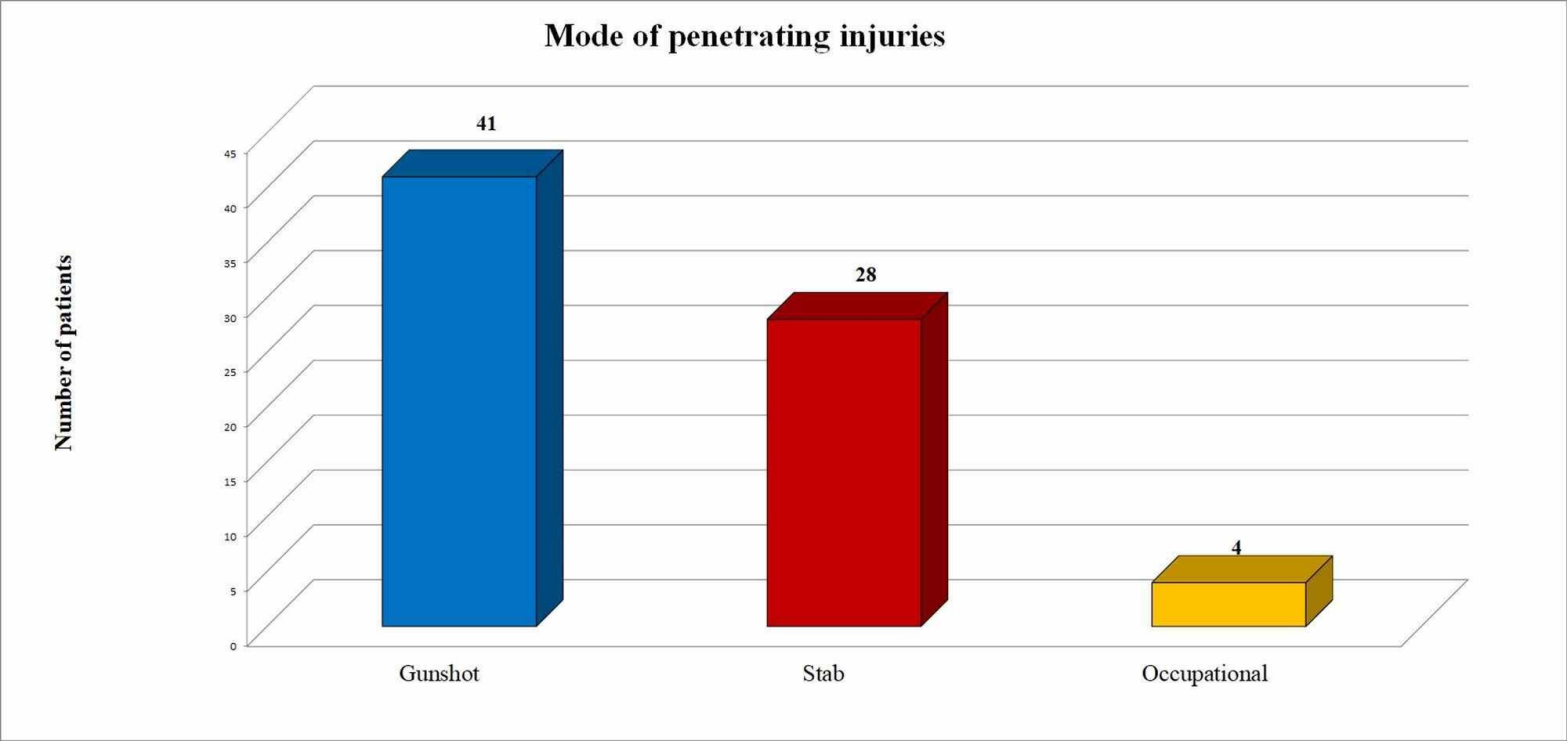
Different modes of penetrating injuries

In our study there were 126 (63.3%) patients with blunt traumas and 73 (36.6%) patients with penetrating traumas. Among penetrating trauma, gunshot injuries (n=41; 56.2%) were the most common, followed by stab wounds (n=28, 38.3%) and occupational injuries (n=4, 5.4%) as shown in Figure 1.

**Figure 2:**
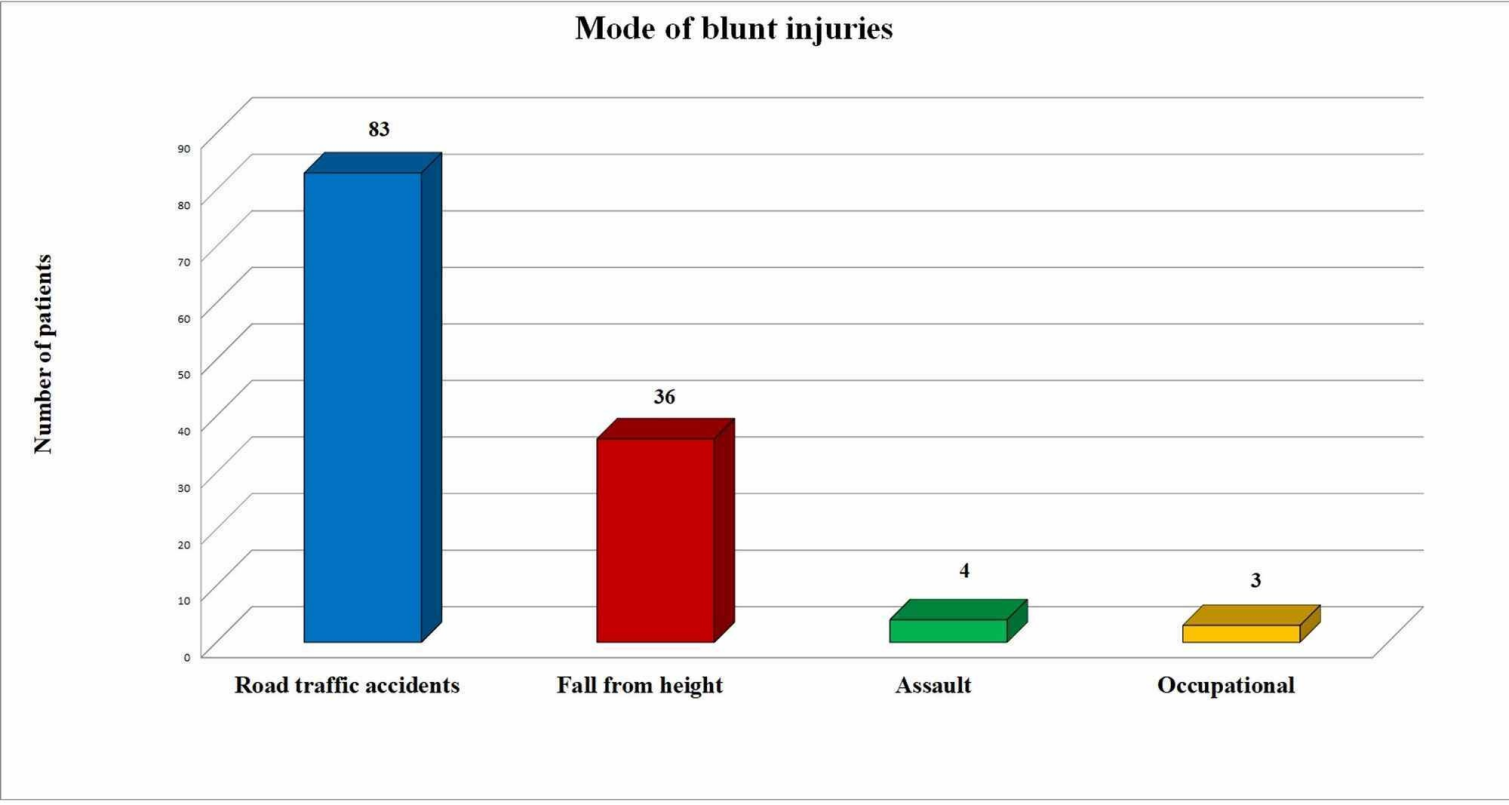
Different modes of blunt injuries

Among blunt traumas, RTAs (n=83; 65.8%) were frequently identified, followed by fall from height (n=36, 28.5%), assault (n=4, 3.2%) and occupational injuries (n=3, 2.4%) as shown in Figure 2.

Motor bike accidents were the most frequent mode of RTA (n=38; 45.7%). There were 21 (25.3%) car-related accidents including car collision, fall from car, and hit by car, and 13 (15.6%) rikshaw related accidents. There were 11 (13.2%) bus-related accidents including bus collision, fall from bus, and hit by bus. Characteristics of all patient injuries including mode of injuries, diagnosis, and associated thoracic and non-thoracic injuries are summarized in Table 1.

**Table 1:** Characteristics of Injuries (N=199)

Characteristics of Injuries	Frequency (%)
MODE OF INJURY	
Blunt	126 (63.3%)
Penetrating	73 (36.6%)
DIAGNOSIS AT THE TIME OF ADMISSION	
Hemothorax	73 (36.7%)
Hemopneumothorax	57 (28.8%)
Pneumothorax	40 (20.1%)
Multiple rib fracture	15 (7.5%)
Miscellaneous	14 (7.0%)
Flail chest	12 (6.0%)
Tension Pneumothorax	2 (1.0%)
ASSOCIATED THORACIC INJURIES	
Rib fracture	117 (58.8%)
Lung contusion	67 (33.7%)
Subcutaneous emphysema	32 (16.1%)
ASSOCIATED NON THORACIC INJURIES	
Bone fractures	102 (51.2%)
Abdominal injuries	32 (16.1%)
Head injuries	23 (11.6%)
Maxillofacial injuries	9 (4.5%)

Among bone fractures, femur (n=39) and clavicle (n=37) were the most common fractured bones. Among 23 patients with associated head injury, 16 (8.0%) patients had traumatic brain injury (TBI) including diffuse axonal injury (DAI) and extra dural hemorrhage (EDH). Of 32 (16.1%) patients with abdominal injuries, liver (n=16,50%) and spleen (n=12, 37.5%) were most commonly involved. The mean time interval between arrival at the ER and medical/surgical intervention was 15.9 ± 12.5 minutes (range: 5-55 minutes).

Surgical intervention included tube thoracostomy in 166 (83.4%), wound repair and debridement in 23 (11.6%), laparotomy in 21 (10.5%), craniotomy in 10 (5.0%), thoracotomy in seven (3.51%), removal of foreign body/bullet in two (1.0%) and conservative management in 24 (12.1%) patients. Out of 166 patients (83.4%) with tube thoracostomy, 119 (71.7%) patients did not require any other surgical intervention and 47 (28.3%) required more than one surgical procedure due to associated non-thoracic injuries. In 16 (69.5%) patients with wound repair, tube thoracostomy was performed. Seven patients required thoracotomy; their indications are summarized in Table 2.

**Table 2:** Indications of thoracotomy (n=7)

S. No.	Indication	Intraoperative Findings	Mode of Trauma
1.	Left Diaphragmatic hernia with multiple rib fracture	Large defect ( 10x10 cm ) with herniated stomach and spleen	Blunt
2.	Unstable sternum with Hemopneumothorax	Sternal body fracture with multiple rib fracture	
3.	>1500 ml blood loss stat	Ruptured Left ventricle and pericardium	
4.		Superior vena cava laceration	Penetrating
5.		Intercostal vessel bleeding	
6.	250ml/hr for more 3 hours	Intercostal vessel bleeding	
7.		Superior vena cava laceration with lung laceration	

The mean blood loss was 169.4 ± 256.5 ml (range: 0-1600 ml). Transfusion of blood products was required in 129 (64.8%) patients. Average numbers of blood products transfused were 2.73 + 1.72.

Mechanical ventilation was needed in 57 (28.6%) patients, 44 patients had blunt trauma (p=0.01), 22 with hemothorax (p<0.001), rib fractures were seen in 40 (p=0.03), 20 patients with abdominal injuries, and 22 with head injury (p<0.001). In patients on ventilatory support, surgical interventions included tube thoracostomy in 56 (p <0.001), thoracotomy in five (p=0.03), other non-thoracic procedures in 30 (p<0.001). MV was also related with those having four or more rib fractures (p=0.02).

The mean duration of hospital stay was 4.4 ± 2.5 days (range: 1-14 days). The mortality rate in our study was 11.1% (n=22). Among these patients, 19 (86.4%) had associated non-thoracic injuries and only three (13.6%) with thoracic injury without any other associated non-thoracic injury (p>0.05). Out of three, one had flail chest with acute respiratory distress syndrome (ARDS) and two patients had massive hemothorax for which they underwent thoracotomy.

Mortality was seen in 22 (11.05%) patients. The predictors of death include higher mean blood loss (365.9 ± 460.6 ml), MV (p=0.001), hemothorax, and associated head injury (p≤0.05). Characteristics of the patients stratified according to their outcome are presented in Table 3.

**Table 3:** Stratification of patient characteristics according to their outcome (N=199)

Variables	Outcome		P value
	Discharge (n=177)	Death (n=22)	
Mode of injury			
Penetrating trauma	69 (38.9%)	4 (18.2%)	0.05
Blunt trauma	108 (61.0%)	18 (81.8%)	
Diagnosis at the time of admission			
Hemothorax	59 (33.3%)	14 (63.6%)	0.005
Hemopneumothorax	52 (29.4%)	5 (22.7%)	0.51
Pneumothorax	38 (20.9%)	2 (13.6%)	0.17
Flail chest	10 (5.9%)	2 (9.1%)	0.52
Multiple rib fracture	15 (8.5 %)	0	NA
Tension Pneumothorax	1 (0.5%)	1 (4.5%)	0.07
Associated thoracic injuries			
Rib fracture	100 (56.5%)	17 (77.3%)	0.06
Lung contusion	62 (35.0%)	5 (22.7%)	0.19
Subcutaneous emphysema	30 (16.9%)	2 (9.1%)	0.34
Associated non-thoracic injuries			
Bone fractures	85 (48.0%)	14 (63.6%)	0.07
Abdominal injuries	24 (13.5%)	6 (27.3%)	0.09
Head injury	13 (7.3%)	10 (45.5%)	<0.001
Maxillofacial injury	8 (4.5%)	1 (4.5%)	0.99
Surgical procedures			
Tube thoracostomy	145 (81.9%)	21 (95.5%)	0.10
Thoracotomy	5 (2.8%)	2 (9.09%)	0.13
Others	53 (29.9%)	10 (45.5%)	0.14
Mechanical ventilation	36 (20.3%)	21 (95.5%)	<0.001
Mean blood loss (ml)	144.9 ± 207.9	365.9 ± 460.6	<0.001

## Discussion

Traumatic thoracic injuries are commonly blunt in nature and affect middle-aged men (30-60 years). Males are more prone as they are mobile and engaged in high-risk activities [4]. The majority of BCIs are a result of RTAs; other common causes include fall from height, assault, and occupational injuries [2-4]. Similar male predilection and involvement of young to middle-aged individuals has also been reported in older Pakistani reports [6,10,11]. In a country like Pakistan, where most of the population behind the wheel is a middle-aged male, the socioeconomic implications of RTAs and subsequent critical injuries are devastating. Blunt traumas may be fatal in case of associated lung injuries or polytrauma [4,8,9]. Among RTAs, motorbike accidents were most frequently encountered. In low-to-middle-income countries, motorbike accidents are a severe problem due to various reasons such as inexperienced bikers, disregard for safety measures, over speeding, overloading bikes with multiple passengers, and road conditions such as narrow, non-maintained, and poorly illuminated at night [12,13]. Penetrating trauma, in this study, was most commonly as a result of gunshot (56.2%) and stab (38.3%) wound. Most international data has reported stab wounds to be more common than gunshot injuries (93.7% vs. 5.94%) [3,14]. In some local studies, stab wounds have been more frequently encountered and in others gunshot wounds [10,11].

We reported hemothorax, hemopneumothorax, and pneumothorax as the common primary thoracic injuries and rib fractures and lung contusions as the common associated thoracic injuries. Hemothorax was related to more adverse outcomes such as MV (p=0.001) and mortality (p=0.005) also reported in other studies [7]. Hemothorax leads to hypovolemic shock pertaining to decrease organ perfusion and further assisted ventilation [15]. Hemothorax in blunt trauma may be due to rib fractures, laceration in lung parenchyma, and injuries to major thoracic and intercostal arteries [2], as is the case in our study.

Rib fractures were associated with MV only (p=0.03). With an increase in number of rib fractures the need for MV also increases. We saw rib fracture of four and more to be associated with MV (p=0.02). Shulzhenko et al. stated that increased number of rib fractures is associated with increase in mechanical ventilation time [16]. Although we did not find any correlation of rib fractures with mortality (p=0.06), Yadollahi et al. in their work reported more than thrice the risk of mortality in blunt trauma patients with rib fractures. Their mortality risk was not associated with hemothorax (p≥0.05) [4] as in our study. Literature has reported varying frequencies of hemothorax, hemopneumothorax, pneumothorax, rib fractures, and lung contusions in patients with thoracic trauma [6,11,17,18]. The frequencies may vary drastically depending on the extent of the injury.

In non-thoracic associated injuries bone fractures - especially femur and clavicle - and injury to abdominal viscera - liver and spleen - was common in our study. Bone fractures were the most frequently encountered associated injury. It did not affect the outcome and was not associated with MV (p=0.40) or mortality (p=0.07). Abdominal injuries were associated with MV (p=0.001); and head injuries, although, not as common, were associated with both MV and morality (p=0.001). Associated extremity and visceral injuries have been commonly reported in the literature [10,17,19]. Association of non-thoracic injuries has been a predictor of mortality in these patients as explained in the literature [20,21]. There is twice the risk of mortality in patients with head injuries associated with thoracic trauma [18].

Tube thoracostomy was utilized for management of most of our patients (83.4%). In the literature, tube thoracostomy was performed in 45-89% patients [3,6,17,21]. Very few of our patients required thoracotomy (3.5%). Literature also reports the need for thoracotomy in a minority of patients (5-9%) [3,6,16,20]. Only a small proportion of cases were managed conservatively (12.1%); the proportion of patients being managed conservatively was higher in the literature (33-38%) [3]. Both tube thoracostomy and thoracotomy were related to MV (p=0.001 and 0.03 respectively).

We managed all of our flail chest patients (n=12; 6%) non-surgically as according to The Eastern Association for the Surgery of Trauma (EAST) guidelines. Surgery should only be reserved for patients who cannot be weaned off from the ventilator [22]. Nine patients with flail chest needed MV, out of which one died secondary to ARDS.

El-Menyar et al. proposed that thoracotomy increased the risk of death by 2.3 times but the study failed to provide any significant statistical correlation. The same results were seen in our study (p=0.13) [18]. The overall mortality rate in our study was 11.1%, similar to a previous report of 12% [6]. With improved risk stratification, mortality can further be reduced. Literature has reported a mortality rate of 1-22% for traumatic thoracic injuries [3,10,11,17,18,20,21,23].

This is the first study to present the pattern and outcome of traumatic thoracic injuries from Jinnah Postgraduate Medical Center, Karachi. This study is unique in reporting for the first time the data from the largest tertiary care institute in south Pakistan. It has helped us document the burden of thoracic trauma in a large multidisciplinary setup even in the duration in which it was studied. It identifies the outcome of thoracic injuries and factors which determine the outcome. However, this study has its limitations too. It cannot generalize the situation of the entire country as it was based on one institution only. Its mortality report is only in-hospital and it did not include post-discharge morbidity or mortality.

## Conclusions

Traumatic thoracic injuries are a preventable cause of mortality in adults. In our population, RTAs, gunshots, and stabs remain a common cause. Tube thoracostomy is frequently needed. Thoracotomy and mechanical ventilation for managing chest injuries are not uncommon. Severity of injury and adverse outcome is determined by the presence of hemothorax, associated abdominal, and head injuries.

In the case of an event, the emergency department team and the team of specialized thoracic surgeons must come together to manage these critical patients with utmost care and vigilance. Favourable outcome can be attained with proper care and management.
